# Dermatological Manifestations of Disseminated Bacillus Calmette-Guerin Infection in the Intensive Care Unit

**DOI:** 10.7759/cureus.67586

**Published:** 2024-08-23

**Authors:** Quentin Saroléa, Mathieu Ziraldo, Lucie Pothen, Leo-Paul Secco, Christine Collienne

**Affiliations:** 1 Critical Care Medicine, Cliniques Universitaires Saint-Luc, Brussels, BEL; 2 Dermatology, Cliniques Universitaires Saint-Luc, Brussels, BEL; 3 Internal Medicine, Cliniques Universitaires Saint-Luc, Brussels, BEL; 4 Dermatopathology, Cliniques Universitaires Saint-Luc, Brussels, BEL

**Keywords:** ziehl-nielsen staining, wade-fite staining, critical care, sepsis, livedo racemosa, purpura, bacillus calmette-guerin infection

## Abstract

We report a case of disseminated Bacillus Calmette-Guerin (BCG)-itis with zosteriform skin eruption, purpura, and livedo racemosa in a 70-year-old critically ill patient who has a history of in situ bladder carcinoma treated with intravesical BCG instillations for the last three years. He presented with fever, fatigue, and a painful lesion on his back, initially diagnosed as herpes zoster. Despite antiviral treatment, he exhibited persistent fever, an inflammatory syndrome, and mild liver enzyme elevation. Initial imaging revealed findings consistent with pneumonia, for which antibiotics were prescribed with no improvement. A subsequent fluorodeoxyglucose (FDG) PET scan identified hypermetabolic lesions in the liver, prompting a biopsy that showed non-caseating granulomas. Skin biopsies from the zosteriform papular eruption on the back and purpura with livedo racemosa on the right foot revealed non-caseating granulomas. Specific Wade Fite staining performed on skin biopsies indicated evidence of mycobacterial infection. Additionally, cultures and Ziehl-Nielsen staining of blood and bone marrow confirmed *Mycobacterium bovis* infection, establishing the diagnosis of disseminated BCG-itis. Treatment with rifampicin, ethambutol, and moxifloxacin, and a later switch to isoniazid, along with corticosteroids, resolved the skin lesions and improved the patient's condition. This case underscores the diagnostic challenges and the importance of considering disseminated BCG-itis in patients treated with prior intravesical BCG instillations for in situ bladder carcinoma presenting with persistent fever, multi-organ involvement, and diverse skin manifestations including zosteriform papules, purpura, and livedo racemosa.

## Introduction

Disseminated Bacillus Calmette-Guérin (BCG)-itis is an uncommon complication in patients treated with BCG intravesical administration for in situ urothelial carcinoma of the bladder, as well as in those who have received the BCG vaccination for tuberculosis protection [1]. It is estimated that approximately 1% of patients who receive intravesical BCG therapy may develop BCG-related infections [2]. Although local complications of vesical instillations mostly consist of cystitis and haematuria, systemic presentations are insidious and atypical, varying with specific organ involvement, and often leading to delays in diagnosis and treatment [1]. Symptoms related to BCG-itis may begin within hours, days, weeks, and even years after the last BCG dose [3]. Cutaneous lesions in the context of BCG-itis are not well described, and to our knowledge, manifestations of such lesions in disseminated BCG-itis have not yet been reported in the literature. Risk factors for BCG-itis include acquired or congenital immunosuppression, diabetes mellitus, genetic susceptibility, recent bladder trauma, subcutaneous injection, and age over 70 [4]. Diagnosing disseminated BCG-itis is challenging due to the absence of diagnostic criteria and the rarity of isolating *Mycobacterium bovis*. Prognosis varies depending on factors such as age, comorbidities, and specific organ involvement [4]. Antimycobacterial treatment regimen and duration remain unclear, but mostly includes isoniazid, rifampicin, and ethambutol for two months, followed by a combination of isoniazid and rifampicin for seven months [5]. 

## Case presentation

**Figure 1:**
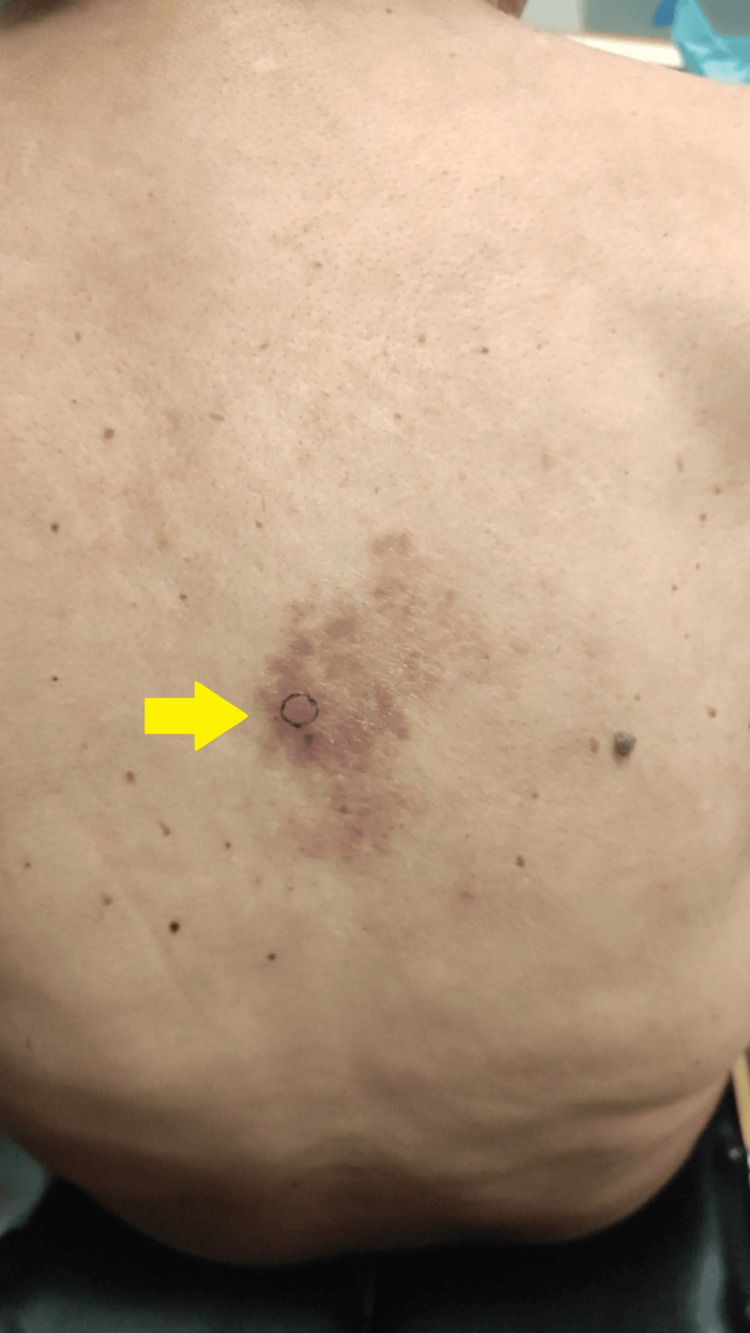
Lesions on the back of the patient The yellow arrow points to unilateral zosteriform papules.

A 70-year-old man presented at the emergency department experiencing low-grade fever, fatigue, and a painful skin lesion on the right laterothoracic area of his back located on the fourth dermatome (Figure 1). A diagnosis of herpes zoster was made, and acyclovir treatment was initiated. The patient returned two weeks later with a persistent fever accompanied by an inflammatory syndrome and a persistent skin lesion on his back, despite treatment. 

The patient’s relevant medical history includes active smoking, chronic obstructive pulmonary disease (COPD), coronary artery disease, atherosclerosis obliterans of the lower limbs, diabetes mellitus, obstructive sleep apnea, and in situ bladder carcinoma, which was treated with intravesical BCG instillations over three years. The last instillation occurred five months before admission. The patient was born in Iran and relocated to Belgium 40 years ago, with no history of tuberculosis, BCG vaccination, or recent travel. One month before admission, the patient underwent percutaneous transluminal coronary angioplasty (PTCA) of the left main coronary artery for a non-ST-segment elevation myocardial infarction (NSTEMI).

Initial laboratory findings revealed elevated C-reactive protein (55.4 mg/L), normal white blood cell count (4.05 x 10³ cells/mm³), normal platelet count (163 x 10³ cells/mm³), mildly decreased glomerular filtration rate (87 mL/min/1.73 m²) mildly elevated serum aminotransferases (aspartate aminotransferase (ASAT): 93 U/L; alanine aminotransferase (ALAT): 70 U/L), elevated alkaline phosphatase (349 U/L), and normal total serum bilirubin levels (1.0 mg/dL) (Table 1).

**Table 1:** Laboratory findings T4: Thyroxine

Parameter	Unit	Normal values
Haemoglobin	13.1 g/dL	13.3-16.7.0 g/dL
White blood cell count	4.05 x 10^3 ^cells/mm^3^	4-10 x 10^3^ cells/mm^3^
Platelet count	163 x 10^3^ cells/mm^3^	130-400 x 10^3^ cells/mm^3^
Prothrombin time	13.3 seconds	9.35 – 14. 30 seconds
International normalized ratio	1.20 seconds	0.80 – 1.20 seconds
Activated partial thromboplastin time	32. 5 seconds	25.1 – 36.5 seconds
Fibrinogen	334 mg/dL	150 - 450 mg/dL
C-reactive protein	55.4 mg/L	0.0-5.0 mg/L
Sodium	132 mmol/L	135-145 mmol/L
Potassium	4.6 mmol/L	3.5-5.0 mmol/L
Chloride	99 mmol/L	97-107 mmol/L
Bicarbonate	20.2 mmol/L	22-29 mmol/L
Glomerular filtration rate	87 ml/min/1.73m²	> 60ml/min/1.73m²
Creatinine	0.89 mg/dL	0.6-1.3 mg/dL
Calcium	2.26 mmol/mL	2.2 – 2.55 mmol/L
Phosphorus	0.83 mmol/L	0.81 – 1.45 mmol/L
Albumin	38 g/dL	35 -52 g/dL
Total bilirubin	1.0 mg/dL	0.20-1.20 mg/dL
Aspartate aminotransferase	93 U/L	18-48 U/L
Alanine aminotransferase	70 U/L	10-40 U/L
Alkaline phosphatase	349 U/L	40-130 U/L
Creatine kinase	100 U/L	20 – 200 U/L
Thyroid-stimulating hormone	0.74 mU/L	0.27 – 5.00 mU/L
T4 free	14.5 pmol/L	12.0 – 22 pmol/L

**Figure 2:**
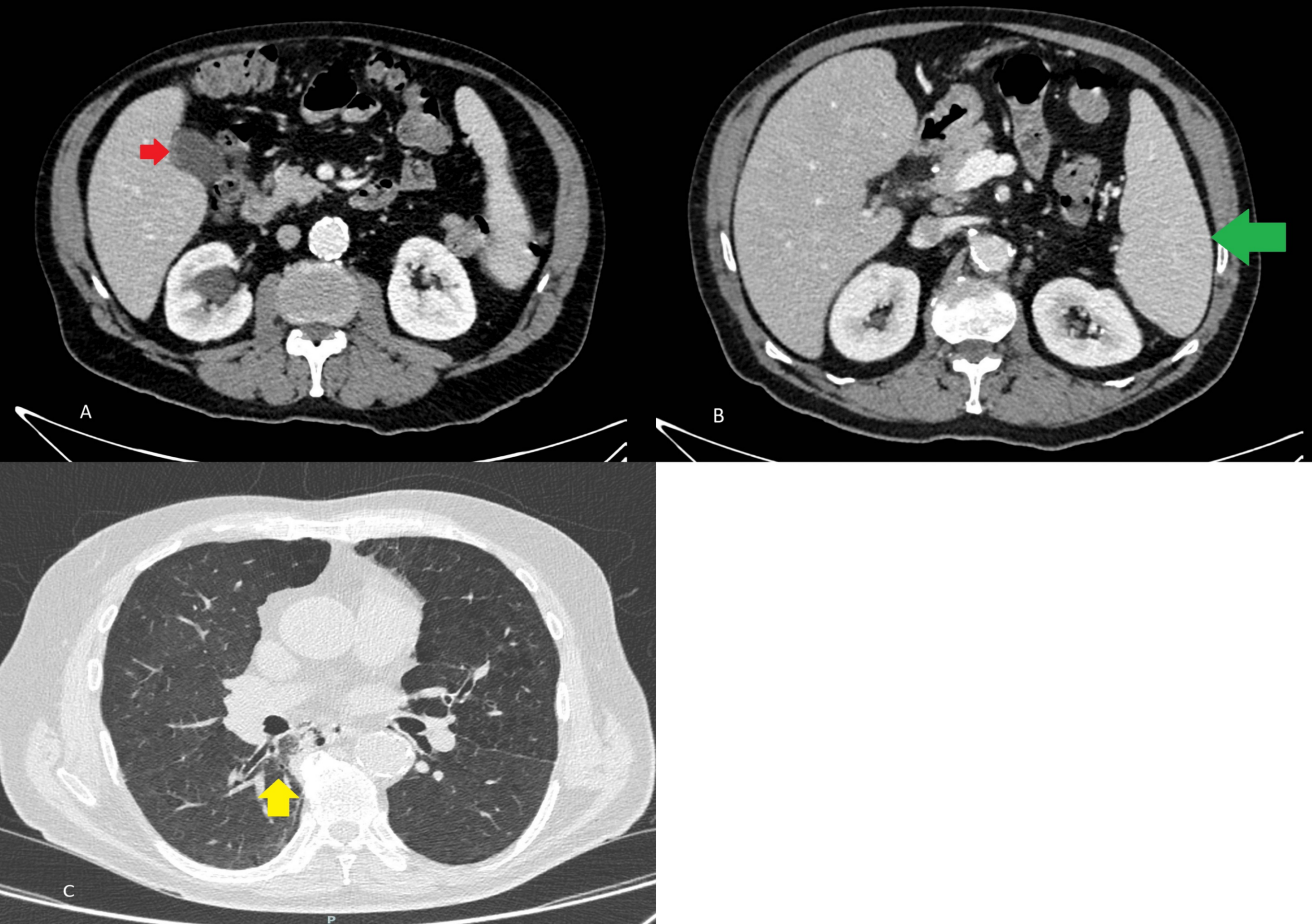
Contrast-enhanced CT of the abdomen A and B: Axial sections of the venous phase show edema of the gallbladder wall without signs of cholecystitis (red arrow) and splenomegaly (green arrow); C: The thorax axial section shows consolidation of the right lower lobe (yellow arrow)

An ultrasound of the bile ducts and a contrast-enhanced abdominal CT scan showed no signs of cholecystitis or liver abnormalities. However, splenomegaly and edema in the gallbladder wall were observed. Additionally, a thoracic CT scan revealed consolidation in the lower lobe of the right lung, suggesting pneumonia (Figure 2). The patient was empirically treated with a seven-day course of cefuroxime 1500 mg three times per day for community-acquired pneumonia; however, there was no improvement.

Nine days after admission, the patient experienced abdominal pain, and an abdominal CT scan showed duodenal perforation requiring surgical duodenal repair. Subsequently, the patient was admitted to the intensive care unit. Microbiological results of intraoperative samples revealed *Candida albicans* and *Enterococcus faecalis*, and he was treated for secondary peritonitis with amoxicillin 2 g four times daily and fluconazole 400 mg once daily for 10 days. Despite adequate antimicrobial treatment, the fever and inflammatory syndrome persisted. Further investigations were therefore carried out.

Aerobic, anaerobic, fungal, and mycobacterial blood cultures were all negative. The urine culture was negative. Viral serologies (hepatitis B virus (HBV), hepatitis C virus (HCV), HIV, cytomegalovirus (CMV), Epstein-Barr virus (EBV), and syphilis) were also negative, as were serologies testing for *Rickettsia* and *Brucella* disease. Finally, autoimmune serologies, including antinuclear antibody (ANA), antineutrophil cytoplasmic antibody (ANCA), anti-*Saccharomyces cerevisiae* antibody (ASCA), myeloperoxidase (MPO) antibody, proteinase 3 (PR3) antibody, and glomerular basement membrane (GBM) antibody, were all negative.

**Figure 3:**
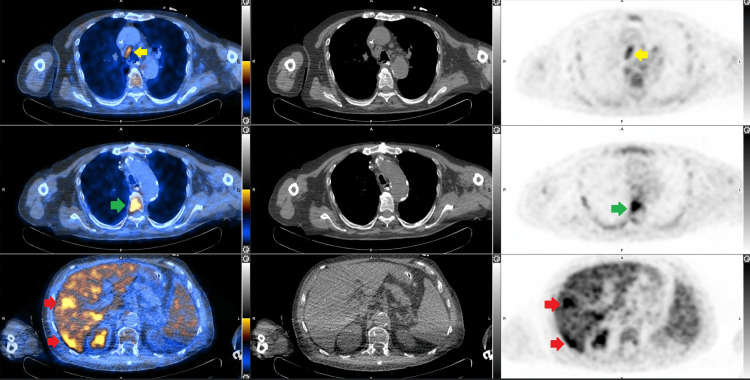
Fused F-18-FDG PET/CT, CT scan, and F-18-FDG PET of the abdomen and thorax The axial sections show hypermetabolic mediastinal lymph nodes (yellow arrow), hypermetabolic activity at the T5 vertebral body (green arrow), and hypermetabolic activity in the right liver (red arrows). F-18-FDG: Fluorine-18 fluorodeoxyglucose

**Figure 4:**
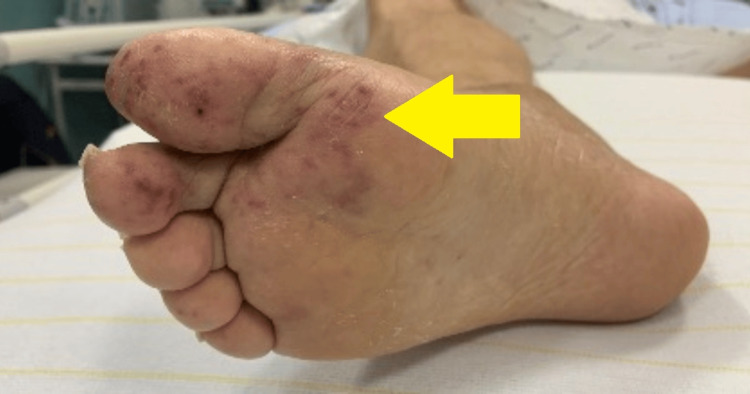
Lesions on the right foot The yellow arrow points to livedo racemosa and palpable purpura on the patient's foot.

Further etiological investigations revealed no signs of infective endocarditis on both fundus and transoesophageal echocardiography. An FDG PET did not reveal any signs of infective endocarditis or lymphoma. However, it identified hypermetabolic lesions in the liver, as well as in the fourth and fifth thoracic vertebrae, and bilateral hypermetabolic pleuropneumopathy with reactive mediastinal lymph nodes (Figure 3). An MRI was carried out on the thoracic vertebrae, which showed no evidence of an infectious lesion. A bone marrow and liver biopsy were performed. Due to the persisting nature of the previously described skin lesion on the back and the appearance of livedo racemosa and palpable purpura on both hands and feet, skin biopsies were also performed (Figure 4).

**Figure 5:**
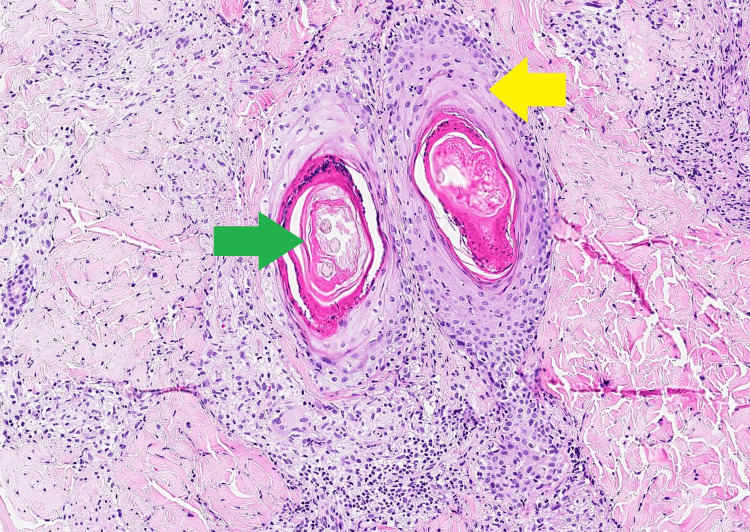
Skin biopsy from right foot with hematoxylin and eosin staining at 100x magnification Green arrow: Capillary thrombosis, Yellow arrow: Granulomatous vasculitis with giant cells

**Figure 6:**
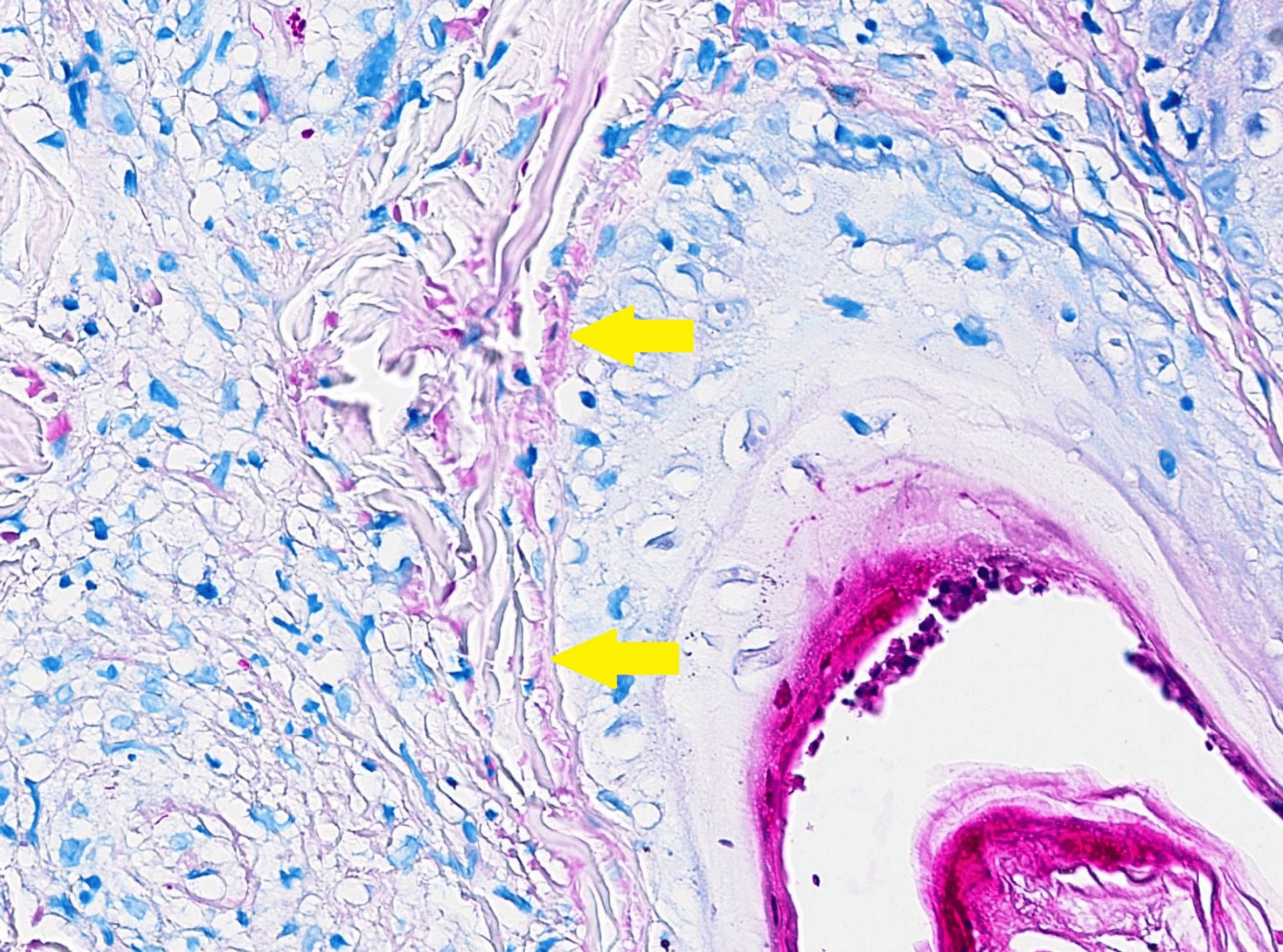
Skin biopsy from the right foot with Wade-Fite staining at 330x magnification Yellow arrows: *Bacillus*

Histological analysis of the skin biopsy samples from the back lesion, taken on day 2 after admission, revealed perifollicular granulomas with epithelial necrosis, without clear cytopathic effects. This pathological pattern was consistent with post-zoster granulomas, where the involvement was limited to adnexal structures. A second examination on day 27 disclosed rod-shaped structures with Wade-Fite staining, which were also described in the biopsy samples from the patient’s right toe. Additionally, the biopsy of the right toe revealed non-caseating granuloma. These findings collectively indicated a high likelihood of mycobacterial dermatitis and vasculitis (Figures 5-6). 

Furthermore, culture and Ziehl-Nielsen staining of the bone marrow (day 15) and blood samples (days 15 and 20) were positive for *M. bovis*. Finally, the liver biopsy revealed numerous necrotizing epithelioid granulomas, although the polymerase chain reaction (PCR) for *Mycobacterium tuberculosis *and culture were negative.

Treatment was started with rifampicin 600 mg once a day, ethambutol 1200 mg three times a week, and moxifloxacin 400 mg daily on day 20 of admission, along with methylprednisolone 0.5 mg/kg/day for 37 days. On day 53, following confirmation of widespread *Mycobacterium* infection, moxifloxacin was switched to isoniazid 300 mg/day, aiming to shorten the overall treatment duration.

The skin lesions on the back, hands, and feet resolved within three weeks after the initiation of antimycobacterial treatment. Additionally, a follow-up FDG PET scan performed on day 70 after treatment revealed a reduction in hypermetabolic lesions in the liver and lungs. Furthermore, there were no new positive blood cultures for *M. bovis*.

Despite receiving adequate antimycobacterial treatment, the patient died seven months after being diagnosed with disseminated BCG-itis. The cause of death was a complete upper airway obstruction, which did not appear to be directly related to the initial diagnosis. Throughout his hospital stay, the patient experienced multiple complications, including recurrent urinary tract infections caused by multidrug-resistant bacteria, prolonged mechanical ventilation, multiple episodes of respiratory failure, acute kidney injury requiring hemofiltration, and a tracheostomy that lasted for a total of six months.

## Discussion

The BCG is a live attenuated bacterium derived from *M. bovis* that provides immunity against various strains of *Mycobacterium*. It has been used as a vaccine against *M. tuberculosis* infection for over a century [6].

The European Association of Urology recommends transurethral resection followed by intravesical instillation of BCG for the treatment of non-muscle-invasive bladder cancer (NMIBC) [7]. Intravesical BCG immunotherapy has demonstrated a long-lasting benefit in preventing NMIBC recurrence and has been shown to improve overall survival over time. Additionally, it has proven effective in reducing the risk of disease progression and metastasis [7].

The mechanisms by which BCG immunotherapy mediates tumor immunity are complex and not fully understood. It is likely that BCG therapy involves a combination of direct interaction between BCG and urothelial or bladder cancer cells, activation of an innate immune response, and initiation of BCG-specific and tumor-specific T-cell immunity [8]. Recent studies suggest that 'trained immunity,' which refers to epigenetic changes in innate immune cells after repeated BCG installations, may play a role in the antitumor effect of BCG in bladder cancer. Regarding adaptive immunity, the induction of activation and differentiation into a T helper 1 (Th1) profile upon presentation of BCG antigens in the urothelium has also been proposed as a mechanism leading to a favorable clinical response to BCG [8,9].

Bacillus Calmette-Guerin-itis remains an uncommon yet potentially severe complication of BCG instillation, with disseminated BCG-itis observed in approximately 1% of cases [2]. Moreover, around 25% of affected patients required admission to the intensive care unit in this case series of 22 patients [10].

Clinical features associated with BCG-itis include both localized and disseminated forms. The localized form may present with transient fever, mictalgia, and pollakiuria and typically occurs within weeks or months after the last exposure to BCG [3,11]. In disseminated BCG-itis, which may manifest months or even years after exposure, systemic symptoms such as persistent fever, night sweats, weight loss, generalized lymphadenopathy, and, in severe cases, septic shock, may occur. Organ dysfunction can be highly variable, leading to diverse clinical presentations depending on the affected organ. The most commonly encountered manifestations involve the liver, lungs, vasculature, muscles, osteoarticular system, and bone marrow [3]. 

The mortality rate in cases of BCG-itis varies, depending on factors such as age, immunosuppression, and comorbidities. According to a Spanish series of 282 patients, the mortality rate is approximately 7.4% for patients over 65 years old and around 2% for those under 65 years old. Additionally, the mortality rate is approximately 10% for the miliary form and approximately 16% for cases with vascular involvement [4]. Long-term complications were observed in 7.4% of cases, with the most frequent issues being loss of visual acuity, arthralgia, and end-stage kidney disease [4].

There is uncertainty regarding the best antimycobacterial treatment and duration in disseminated BCG-itis, as there is a lack of prospective trials. Due to the inherent resistance to pyrazinamide of *M. bovis*, antimycobacterial therapy usually involves a combination of isoniazid, rifampicin, and ethambutol. The Infectious Diseases Society of America (IDSA) subsequently recommends a regimen of isoniazid, rifampicin, and ethambutol for two months, followed by treatment with isoniazid and rifampicin for seven months [5]. Treatment regimens that incorporate fluoroquinolones have also been documented, especially in situations where the initial diagnosis remains uncertain or in cases of co-infections requiring a broader antibiotic spectrum, as was the case with our patient [4]. In cases where there is widespread miliary lung involvement and/or respiratory failure, glucocorticoids have been reported to be used alongside antimycobacterial medications with good clinical outcomes in case reports [4].

The skin clinical pattern of BCG-itis, including its topography, distribution, primary lesion, and associated symptoms, is not well defined. Skin involvement tends to be polymorphic and can look like other dermatoses, resulting in limited information within the dermatological literature [12]. Additionally, most of the cutaneous manifestations described in the literature resulted from a BCG vaccination. Specifically, peripheral small vessel vasculitis with palpable purpura and racemosa livedo is not well reported in the literature. Histological findings, PCR analysis on tissue samples, and specific staining techniques are the primary diagnostic tools in this condition. Quantiferon remains usually negative, unlike *M. tuberculosis* infection [13].

The histological appearance of skin biopsies in BCG-itis varies depending on the patient’s immune status. In immunosuppressed patients, biopsies typically show a diffuse infiltrate of plump histiocytes with poorly formed or absent granulomas. The cytoplasm of these histiocytes is often markedly distended by numerous acid-fast *M. bovis *Bacilli*.* In contrast, immunocompetent individuals usually present with multiple epithelioid cell granulomas in skin and lymph node biopsies. These granulomas frequently contain Langhans giant cells and exhibit minimal caseous necrosis, with or without associated suppuration [14]. In comparison, the histopathological features of tuberculosis commonly include epithelioid granulomas with central caseous necrosis, frequently surrounded by Langhans giant cells [15].

Finally, the diagnosis of disseminated BCG-itis relies on a combination of clinical, pathological, and microbiological findings. Histological analysis alone cannot distinguish between infections caused by *M. bovis*, *M. tuberculosis*, or non-tuberculous mycobacteria. Therefore, culture and PCR are essential for the accurate identification of the mycobacterial species involved [14].

This case report presents a patient with disseminated BCG-itis showing unusual skin manifestations, including a zoster-like pattern followed by livedo racemosa, which, to our knowledge, has not been described to date. Our aim is to raise awareness among physicians regarding the subtle and diverse clinical features of disseminated BCG-itis, the related diagnostic challenge, and the limited sensitivity of culture tests. Clinicians should consider this diagnosis in patients who have undergone intravesical BCG instillation and are presenting with persistent fever, elevated inflammatory markers, multi-organ involvement without a formal alternative diagnosis, and varied skin manifestations such as zosteriform papules, purpura, and livedo racemosa.

## Conclusions

Dermatological manifestations of disseminated BCG-itis are not well reported in the literature and may be diverse, including a zosteriform pattern, livedo racemosa, and palpable purpura. Clinicians should consider disseminated BCG-itis in critically ill patients with a history of intravesical BCG instillation who present with fever of unknown origin and multiple organ involvement after initial exclusion of infectious, systemic, and autoimmune diseases. In such patients, when cutaneous manifestations are present, a skin biopsy should be performed, as it can be crucial for confirming the diagnosis and guiding appropriate management.

Initial laboratory findings typically yield unspecific information, often resulting in a delayed diagnosis. Suggestive histopathological features, such as non-caseating histiocytic granulomas and bacilli identified through West-Fite and Ziehl-Neelsen staining, serve as strong indicators when disseminated BCG-itis is suspected. However, these findings alone cannot distinguish BCG-itis from infections caused by other mycobacterial species. A definitive diagnosis is established with positive culture or PCR evidence of *M. bovis* in blood culture or tissue samples.

The best antimycobacterial treatment regimen and duration remain unclear but usually include isoniazid, rifampicin, and ethambutol for two months. This is followed by a combination of isoniazid and rifampicin for seven months. Further evidence is needed to recommend parenteral glucocorticoids in cases of miliary lung involvement and/or respiratory failure.
